# The Clinical, Radiological and Genetic Spectrum of *PLA2G6*-Associated Neurodegeneration: An Experience From a Tertiary Center

**DOI:** 10.5334/tohm.897

**Published:** 2024-08-21

**Authors:** Vikram V. Holla, M. M. Samim, Riyanka Kumari, Debjyoti Dhar, Prashant Phulpagar, Neeharika Sriram, Shweta Prasad, Jitender Saini, Nitish Kamble, Ravi Yadav, Babylakshmi Muthusamy, Pramod Kumar Pal

**Affiliations:** 1 Department of Neurology, National Institute of Mental Health and Neurosciences, Bengaluru, 560029, India; 2 Institute of Bioinformatics, International Technology Park, Bangalore, 560066, India; 3 Manipal Academy of Higher Education, Manipal, 576104, Karnataka, India; 4 Department of Neuroimaging and Interventional Radiology, National Institute of Mental Health and Neurosciences, Bengaluru, 560029, India; 5 Department of Medical Genetics, Kasturba Medical College, Manipal Academy of Higher Education, Manipal, India

**Keywords:** Dystonia, PLAN, PLA2G6, INAD, early-onset Parkinson’s disease, ANAD, HSP

## Abstract

**Background:**

Despite being the second most common type of neurodegeneration with brain iron accumulation, there is limited literature on *PLA2G6*-associated neurodegeneration (PLAN) within the Asian ethnicity, particularly in the Indian context.

**Methods:**

We conducted a retrospective observational study on patients with pathogenic/likely pathogenic *PLA2G6* variants based on exome sequencing.

**Results:**

We identified 26 patients (22 families, 15 males) of genetically-confirmed PLAN with a median age of 22.5 years and age at onset of 13.0 years, encompassing various subtypes: infantile neuroaxonal dystrophy (5/26;19.2%), atypical neuroaxonal dystrophy (3/26;11.5%), dystonia-parkinsonism (5/26;19.2%), dystonia-parkinsonism-myoclonus (n = 4, 15.38%), early-onset Parkinson’s disease (2/26;7.7%), complex dystonia (2/26;7.7%), and complicated hereditary spastic paraparesis (cHSP; 5/26;19.2%). The common initial symptoms included walking difficulty (7/26;26.9%), developmental regression (6/26;23.1%), and slowness (4/26;15.4%). Dystonia (14/26;53.8%), followed by parkinsonism (11/26; 42.3%), was the most common motor symptom. Non-motor symptoms included cognitive decline (12/26;46.2%) and behavioral changes (6/26;23.1%). Neuroimaging revealed cerebellar atrophy in 23/26 (88.5%) patients and claval hypertrophy in 80% (4/5) of INAD patients. Levodopa responsiveness was noted in 12 of 14 patients with parkinsonism/dystonia who received levodopa, and dyskinesia was noted in 10/11 patients. Genetic analysis revealed a total of 19 unique variants in *PLA2G6* gene, of which 11 were novel. Twelve patients harbored the c.2222G>A variant, which is predominantly seen in Asian subpopulations.

**Conclusions:**

The study introduces 26 new patients of PLAN and 12 patients associated with the c.2222G>A variant, potentially forming the most extensive single center series to date. It also expands the phenotypic, neuroimaging, and genotypic spectrum of PLAN.

## Introduction

*PLA2G6*-associated neurodegeneration (PLAN), representing the second most common type of neurodegeneration with brain iron accumulation (NBIA), encompasses a number of unique clinical entities [1]. Depending on the clinical manifestation across different stages of life, these comprise infantile neuroaxonal dystrophy (INAD, NBIA2 A; MIM 256600), atypical neuroaxonal dystrophy (aNAD, NBIA2B; MIM 610217), and adult-onset dystonia-parkinsonism (DP, PARK14, MIM 612953) [1,2]. Diagnosis and subcategorization into distinct subgroups depend on various factors such as clinical symptoms, disease progression, neurophysiological assessments, radiographic studies, and laboratory tests [3]. Establishing a genetic diagnosis for these phenotypes constitutes a definite challenge owing to the complexities of clinical presentation and the rarity of this disease. At the molecular level, the *PLA2G6* gene is situated on chromosome 22 at 22q13.1, and contains 17 exons spanning over 69 kilobases (kb). It encodes the CaI-PLA2 protein, which is pivotal in catalyzing hydrolysis within phospholipids. Mutations in *PLA2G6* disrupt its function of repairing oxidative damage in phospholipid membranes, which affects the membrane integrity and fluidity, thereby contributing to the underlying pathological mechanisms.

Previous large-scale studies have continued to add to the constellation of phenotypes, highlighting the importance of having a high index suspicion despite atypical clinical presentation [1,3]. As the disease continues to evolve, recent studies have revealed the role of ethnicity in dictating the genotypic variants and the clinical phenotype. Against this backdrop, we conducted a retrospective observational single-center study to describe in-depth, the clinical phenotypes and correlate them with the genotype of the patients evaluated so far in our neurology center.

## Methods

### Patients and methods

We performed a retrospective observational study of all patients exhibiting a biallelic pathogenic or likely pathogenic variants in the *PLA2G6* gene based on exome sequencing in the probands. The case records were subjected to detailed data extraction comprising details of age at onset (AAO), age at presentation, first symptom at onset, the spectrum of motor symptoms, movement disorders encountered, non-motor clinical features including cognitive, psychiatric, and additional relevant clinical features, genotype, management strategies including levodopa responsiveness and drug-induced dyskinesia. All patients were classified based on the clinical phenotype into INAD, aNAD, dystonia-parkinsonism (DP), dystonia parkinsonism myoclonus (DPM), early-onset parkinsonism (EOP), complex dystonia and complicated hereditary spastic paraparesis (cHSP) phenotype. The study was approved by the institute’s ethics committee. Written informed consent was obtained from patients for the publication of recorded videos.

### Genetic analyses

The blood samples of patients were subjected to Genomic DNA extraction using QIAamp DNA Blood Mini Kit (Qiagen Germany, #51104). Subsequently, raw reads were aligned to the human reference genome (GRCh37) based on the BMA-mem algorithm. Polymerase chain reaction (PCR) duplicates were removed using the Picard toolkit (https://broadinstitute.github.io/picard/) [4]. The variants were identified based on the Genome Analysis Toolkit (GATK) framework (Broad Institute, Cambridge, MA, USA). Variants were exposed to base quality score recalibration for filtration, following which annotations would be done in the ANNOVAR platform (http://www.openbioinformatics.org/annovar/) [5]. The variants with a minor allele frequency (MAF) >0.01, suggestive of common occurrence in the population, were not included. A comparison of data with the Exome Aggregation Consortium (ExAC), 1000 Genome project, and gnomAD database (https://gnomad.broadinstitute.org/) was performed. Each of the individual sequence variants were tested using PolyPhen-2, Sorting Intolerant from Tolerant (SIFT) webserver, and MutationTaster [6,7,8]. The variants were assigned a pathogenicity coding according to the American College of Medical Genetics and Genomics (ACMG) guidelines into benign, likely pathogenic or pathogenic [9]. Each of these genes was analyzed in the mutation databases of ClinVar for novelty evaluation.

### Review of literature

We conducted a systematic search across the accessible medical database of PubMed, Google Scholar, and Scopus, using the medical subject headings (MeSH): “PLA2G6”, “PLAN”, “INAD”, “ANAD”, “atypical NAD”, “Hereditary Spastic Paraparesis”, parkinsonism”, “young onset Parkinson’s disease”, “early onset Parkinson’s disease”, “dystonia”, and “disorder” to identify all the relevant studies. The articles were subjected to title and abstract screening. A shadow search of the reference articles was done to avoid missing key articles. Our data extraction process was based on identifying unique demographic and clinical parameters, classifying the clinical phenotype, and describing the specific genetic variants so identified. Studies not presented in English or lacking patient details were excluded from consideration.

### Statistical methods

Categorical variables were depicted in terms of frequencies, whereas continuous variables were designated as median values along with their respective interquartile ranges. The initial analysis involved descriptive statistics to examine the demographic, clinical, and neuroimaging parameters. A narrative description encompassing clinical phenotypes and genotypes and their correlation with neuroimaging parameters was made. Statistical calculations were performed using SPSS version 23.0.

## Results

### Demographics

In this retrospective analysis, we analyzed 26 patients (22 families) with genetically confirmed PLAN. Eight of these patients were part of a multicentric publication [3]. There was a male predominance (n = 15, 57.69%) distributed across 22 families. The median age at presentation was 22.5 years (IQR: 6.5–29.0), while the median age at onset (AAO) was 13.0 years (IQR: 2.7–20.5) and the patients presented after a median duration of illness of 3.5 months (IQR: 1.5–10.5). Consanguinity was observed in 53.8% of patients (n = 14), while 42.3% (n = 11) had a positive family history (Table 1).

**Table 1:** Demographic, clinical and radiological features of patients with PLAN.


	TOTAL (n = 26)	INAD (n = 5)	ANAD (n = 3)	DP (n = 5)	DPM (n = 4)	EOP (n = 2)	COMPLEX DYSTONIA (n = 2)	cHSP (n = 5)

				PARKINSONISM (n = 11)				

Gender (Male: Female)	15:11	3:2	2:1	1:4	3:1	1:1	1:1	4:1

Age at presentation, Year (median, IQR)	22.5 (6.5–29.0)	2.0 (1.9–2.7)	7.0 (5.0–8.0)	29.0 (25.5–35.0)	28.5 (25.0–30.5)	29.0	21.0	23.0 (18.0–29.0)

Age at Onset, Year (median, IQR)	13.0 (2.7–20.5)	1.4 (0.75–1.75)	3.0 (2.0–4.0)	15.0 (12.0–22.7)	18.0 (12.0–27.0)	26.5	16.5	18.0 (10.5–20.0)

Duration of illness, month (median, IQR)	3.5 (1.5–10.5)	1.0 (0.50–1.45)	3.0 (2.0–6.0)	14.0 (5.2–20.5)	8.0 (3.3–15.8)	2.5	4.1	10.0 (3.0–11.0)

Consanguinity (n, %)	14 (53.8)	4 (80.0)	0	2 (40)	2 (50.0)	1 (50.0)	2 (100)	3 (60.0)

Positive family history (n, %)	11 (42.3)	0	0	2 (40)	4 (100.0)	0	2 (100)	3 (60.0)

History of Global Developmental Delay	9 (34.6)	4 (80.0)	2 (66.7)	0	0	0	2 (100)	1 (20.0)

Dysmorphism	4 (15.4)	3 (60.0)	1 (33.3)	0	0	0	0	0

**Symptoms at the onset**								

Developmental regression	6 (23.1)	5 (100)	1 (33.3)	0	0	0	0	0

Cognitive decline	3 (11.5)	0	0	0	3 (75.0)	0	0	0

Behavioural symptoms	2 (7.7)	0	0	2 (40)	0	0	0	0

Abnormal Posturing	2 (7.7)	0	0	0	0	0	2 (100)	0

Walking difficulty	7 (26.9)	0	1 (33.3)	2 (40)	0	0	0	4 (80.0)

Slowness	4 (15.4)	0	0	1 (20)	1 (25.0)	2 (100)	0	0

Change in speech	2 (7.7)	0	1 (33.3)	0	0	0	0	1 (20.0)

**Symptomatology**								

Behavioural changes	6 (23.1)	0	0	1 (20)	2 (50.0)	0	0	3 (60.0)

Cognitive decline	12 (46.2)	0	2 (66.7)	2 (40)	4 (100.0)	0	0	4 (80.0)

Drooling of saliva	2 (7.7)	1 (20.0)	0	()	0	0	0	1 (20.0)

Pseudobulbar affect	4 (15.4)	0	0	2 (40)	0	0	0	2 (40.0)

Epilepsy	4 (15.4)	0	1 (33.3)	0	1 (25.0)	0	0	2 (40.0)

**Examination**								

Strabismus	4 (15.4)	3 (60.0)	0	1 (20)	0	0	0	0

Optic atrophy	1 (3.8)	1 (20.0)	0	0	0	0	0	0

Nystagmus	3 (11.5)	2 (40.0)	1 (33.3)	0	0	0	0	0

Gaze restriction	11 (42.3)	2 (40.0)	0	1 (20)	3 (75.0)	0	2 (100)	3 (60.0)

Speech abnormality								

*Mild*	2 (7.7)	1 (20.0)	0	0	0 ()	0	0	1 (20.0)

*Moderate*	7 (26.9)	0	2 (66.7)	2 (40)	1 (25.0)	0	1 (50.0)	1 (20.0)

*Severe*	2 (7.7)	0	1 (33.3)	0	0	0	0	1 (20.0)

*Normal*	15 (57.7)	4 (80.0)	0	3 (60)	3 (75.0)	2 (100)	1 (50.0)	2 (40.0)

Dysarthria types								

*Spastic*	2 (7.7)	0	0	0	0	0	1 (50.0)	1 (20.0)

*Hyperkinetic*	6 (23.1)	1 (20.0)	2 (66.7)	1 (20)	1 (25.0)	0	0	1 (20.0)

*Ataxic*	2 (7.7)	0	0	2 (7.7)	0	0	0	0

*Anarthria*	2 (7.7)	0	1 (33.3)	0	0	0	0	1 (20.0)

*Normal speech*	14 (53.8)	4 (80.0)	0	2 (0)	0	2 (100)	1 (50.0)	2 (40.0)

Tone								

*Rigidity*	12 (46.2)	1 (20.0)	0	5 (100)	4 (100.0)	2 (100)	0	0

*Spasticity*	7 (26.9)	1 (20.0)	1 (33.3)	0	0	0	0	5 (100.0)

*Hypotonia*	5 (19.2)	3 (60.0)	2 (66.7)	0	0	0	0	0

Brisk muscle stretch reflex	17 (65.4)	2 (40.0)	1 (33.3)	2 (40)	4 (100.0)	1 (50.0)	2 (100)	5 (100.0)

Positive Babinski sign	12 (46.2)	2 (40.0)	2 (66.7)	3 (60)	1 (25.0)	0	0	4 (80.0)

Extrapyramidal features								

*Dystonia*	14 (53.8)	0	0	5 (100)	4 (100.0)	0	2 (100)	3 (60.0)

Generalized	12 (46.2)	0	0	5 (100)	3 (75.0)	0	2 (100)	2 (40.0)

Lower limb only	1 (3.8)	0	0	0	1 (25.0)	0	0	0

Finger only	1 (3.8)	0	0	0	0	0	0	1 (20.0)

*Parkinsonism*	11 (42.3)	0	0	5 (100)	4 (100.0)	2 (100)	0	0

*Tremor*	7 (26.9)	0	0	3 (60)	2 (50.0)	0	1 (50.0)	1 (20.0)

*Positive cerebellar signs*	14 (53.8)	4 (80.0)	3 (100.0)	3 (60)	0	0	1 (50.0)	3 (60.0)

*Myoclonus*	6 (23.1)	0	0	()	4 (100.0)	0	0	2 (40.0)

Contractures	11 (42.3)	2 (40.0)	1 (33.3)	3 (60)	2 (50.0)	0	0	3 (60.0)

Gait								

*Dystonic*	11 (42.3)	1 (20.0)	0	5 (10)	0	0	1 (50.0)	0

*Ataxic*	6 (23.1)	2 (40.0)	2 (66.7)	0	2 (50.0)	2 (100)	0	2 (40.0)

*Spastic*	4 (15.4)	1 (20.0)	1 (33.3)	0	0	0	0	2 (40.0)

*Normal*	5 (19.2)	1 (20.0)	0	0	2 (50.0)	0	1 (50.0)	1 (20.0)

EEG (n = 6)								

*Abnormal EEG*	4/6 (66.7)	1/2 (50.0)	1/1 (100)	0	0	0	0	2/2 (100)

Generalized slowing	2/6 (33.3)	0	1/1 (100)	0	0	0	0	1/2 (50.0)

Focal IEDs	2/6 (33.3)	1/2 (50.0)	0	0	0	0	0	1/2 (50.0)

*Normal*	2/6 (33.3)	1/2 (50.0)	0	1/1 (100)	0	0	0	0

Abnormal nerve conduction study	2/10 (20.0)	1/2 (50.0)	0/1	1/4 (25.0)	0	0	0/1	0/2

MRI	n = 25	n = 5	n = 3	n = 5	n = 4	n = 2	n = 2	n = 4

Mineralization								

*Caudate*	9 (34.6)	1 (20.0)	0	3 (60)	2 (50.0)	0	0	3 (75.0)

*Putamen*	8 (30.8)	1 (20.0)	0	3 (60)	1 (25.0)	0	0	3 (75.0)

*GPi*	14 (53.8)	2 (40.0)	2 (66.7)	5 (100)	1 (25.0)	1 (50.0)	0	3 (75.0)

*Substantia nigra*	14 (53.8)	2 (40.0)	2 (66.7)	4 (80)	1 (25.0)	1 (50.0)	0	4 (100.0)

Atrophy								

*Caudate*	4 (15.4)	0	0	1 (20)	2 (50.0)	0	0	1 (25.0)

*Cerebral*	10 (38.5)	1 (20.0)	1 (33.3)	2 (40)	3 (75.0)	0	0	3 (75.0)

*Frontal predominant*	3 (11.5)	0	0	1 (20)	1 (25.0)	0	0	1 (25.0)

*Corpus callosum*	1 (3.8)	1 (20.0)	0	0	0	0	0	0

*Optic nerve*	2 (7.7)	1 (20.0)	1 (33.3)	0	0	0	0	0

*Cerebellar*	23 (88.5)	5 (100)	3 (100.0)	5 (100)	3 (25.0)	1 (50.0)	2 (100)	4 (100)

White matter signal changes	12 (46.2)	2 (40.0)	2 (66.7)	4 (80)	1 (25.0)	0	0	3 (75.0)

Claval hypertrophy	10 (38.5)	4 (80.0)	1 (33.3)	2 (40)	0	1 (50.0)	0	2 (50.0)

Response to Levodopa	12/14 (85.7)	0	0	5/5 (100)	4/4 (100.0)	2/2 (100)	1/2 (50.0)	0/1

Levodopa-induced-Dyskinesia	10/11 (90.9)	0	0	4/5 (80)	4/4 (100.0)	2/2(100)	0	0


aNAD: Atypical neuroaxonal dystrophy; cHSP: Complicated Hereditary spastic paraparesis; DP: Dystonia parkinsonism; DPM: Dystonia parkinsonism myoclonus; EEG: Electroencephalograph; EOP: Early onset Parkinsonism; INAD: Infantile Neuroaxonal Dystrophy; IQR: Inter quartile range.

### Clinical features

#### Symptomatology

At the onset of the disease, patients exhibited diverse symptoms, with difficulty in walking (7/26;26.9%), developmental regression (6/26;23.1%), slowness of movements (4/26;15.4%), cognitive decline (3/26;11.5%), behavioral symptoms (2/26, 7.7%), abnormal posturing (2/26, 7.7%), and changes in speech (2/26, 7.7%) (Table 1). A history of global developmental delay was observed in 34.6% (9/26) of patients, while 15.4% (4/26) exhibited dysmorphism. During the course of the illness, behavioral changes were observed in 6 patients (6/26;23.1%), cognitive decline in 12 patients (12/26;46.2%), epilepsy in 4 patients (4/26;15.4%), drooling of saliva in 2 patients (2/26;7.7%), and pseudobulbar affect and strabismus in 4 patients each (4/26;15.4% each).

#### Extrapyramidal symptoms

Dystonia was evident in 14 patients (14/26;53.8%) of which 12 patients had generalized subtype (12/26;46.2%). Additionally, lower limb-only and finger-only involvement was noted in one patient (1/26;3.8%) each. Parkinsonism was present in 11 patients (11/26;42.3%), and tremor was noted in 7 patients (7/26;26.9%). Myoclonus was noted in 6 patients (6/26;23.1%). Cerebellar signs were present in 14 patients (14/26;53.8%). With regards to gait assessment, 11 patients (11/26;42.3%) exhibited dystonic patterns, 6 (6/26;23.1%) showed ataxic patterns, 4 (4/26;15.4%) displayed spastic patterns, and 5 patients (5/26;19.2%) had a normal gait (Table 1).

#### Other neurological signs

Optic atrophy was found in one (1/26;3.8%), and nystagmus was present in 3 patients (3/26;11.5%). Gaze restriction was a prevalent symptom, affecting 11 patients (11/26;42.3%). In most patients, the upgaze was mildly restricted, and there were no square wave jerks. Vestibulo-ocular maneuverer and optokinetic nystagmus reflex could not be performed in many. Vestibulo-ocular maneuverer was performed in two patients with cHSP phenotype, and the upgaze movement was better, suggesting a possibility of supranuclear type of abnormality. Twelve patients (12/26;46.2%) had abnormal speech, of which 6 patients had hyperkinetic dysarthria (6/26;23.1%), and 2 patients (2/26;7.7%) each had spastic dysarthria, ataxic dysarthria and anarthria respectively. With regards to the severity of dysarthria, the majority had moderate affliction (7/26;26.9%) while 2 patients (2/26;7.7%) each had mild and severe impairment. The majority of patients exhibited abnormal muscle tone, (24/26;92.3%) that manifested either as rigidity (12/26;46.2%), spasticity (7/26;26.9%), or hypotonia (5/26;19.2%). A brisk muscle stretch reflex was evident in 17 patients (17/26;65.4%) while a positive Babinski sign was observed in 12 patients (12/26;46.2%). Contractures were observed in 11 patients (11/26;42.3%).

#### Phenotypic classification

The spectrum of phenotypes comprised of INAD in 5 patients (19.2%), ANAD in 3 patients (11.5%), complex dystonia in 2 patients (7.7%), complex hereditary spastic paraparesis (cHSP) in 5 patients (19.2%), and parkinsonism syndromes that included dystonia-parkinsonism (DP) in 5 patients (9.23%), dystonia parkinsonism myoclonus (DPM) in 4 patients (15.4%), and early-onset parkinsonism (EOP) in 2 patients (7.7%) (Table 1).

### Investigations and management

#### Neuroimaging features

**Figure 1:**
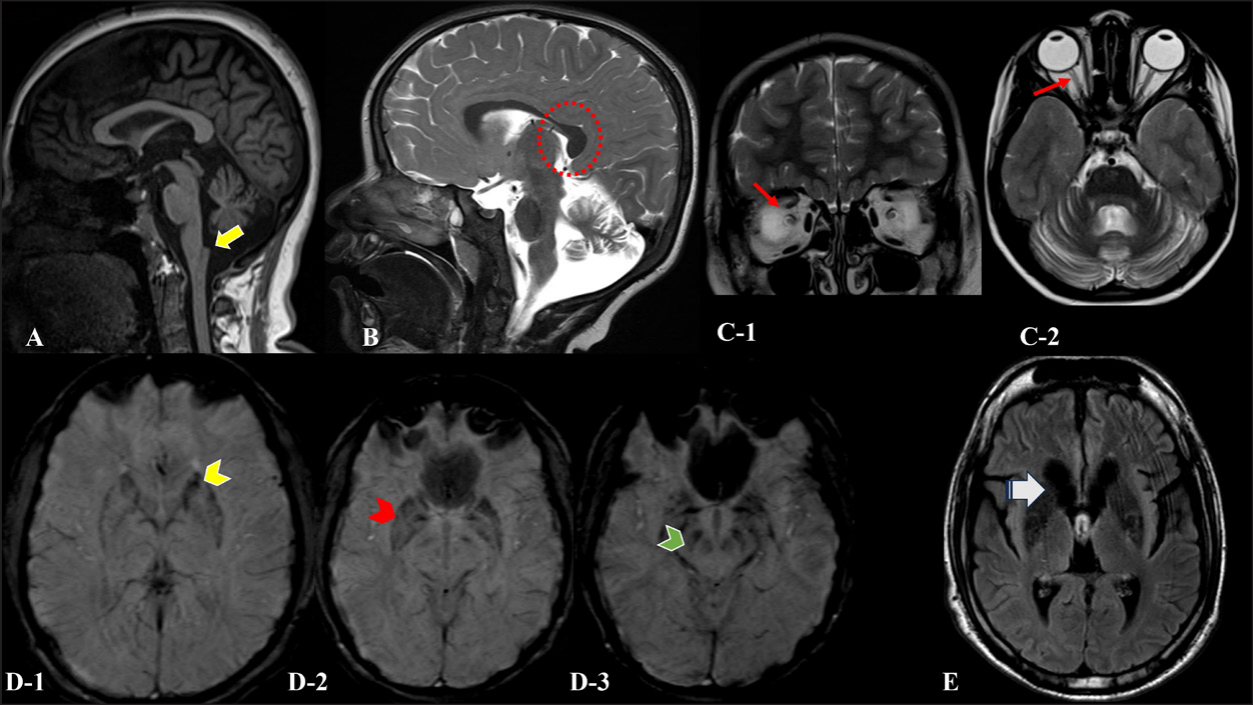
**Magnetic resonance imaging of the brain of selected patients. A:** Patient of early-onset parkinsonism (Patient-22). T1 mid-sagittal image showing claval (gracile tubercle) hypertrophy (yellow arrow). **B:** Patient of infantile neuroaxonal dystrophy (Patient-12). T2 mid-sagittal image showing vertically oriented splenium of the corpus callosum (red dotted circle). **C:** Patient of atypical neuroaxonal dystrophy (Patient-16). T2 coronal (C-1) and axial (C-2) images showing bilateral optic nerve atrophy (red arrow). **D:** Patient of complicated hereditary spastic paraparesis (Patient-13). SWI image showing mineralization of bilateral caudate (D-1, yellow arrowhead), putamen, globus pallidi (D-2, red arrow head), and substantia nigra (D-3, green arrow head). **E:** Patient of complicated hereditary spastic paraparesis (Patient-19). Fluid attenuated inversion recovery (FLAIR) axial image showing atrophy of bilateral caudate and generalized cerebral atrophy (white arrow). It also shows FLAIR hypointensity of bilateral putamen and globus pallidi.

Brain MRI (available for 25 patients) showed mineralization in various areas, including the caudate in 9/25 patients (34.6%), the putamen in 8/25 patients (30.8%), the GPi in 14/25 patients (53.8%), and the substantia nigra in 14/25 patients (53.8%). Atrophy was noted in different brain structures, of which cerebellum was involved most commonly (23/25;88.5%) followed in order by diffuse cerebral (10/25;38.5%), caudate (4/25;15.4%), frontal-predominant atrophy (3/25;11.5%), optic nerve (2/25;7.7%) and corpus callosum (1/25; 3.8%). Other findings included white matter signal changes in 12 patients (12/25;46.2%) and claval hypertrophy in 10 patients (10/25;38.5%). With regards to the individual subtypes, classic claval hypertrophy was observed in 80% (n = 4/5) of patients of INAD followed by ANAD (1/3;33.3%) and DP (2/5;40%). This claval hypertrophy refers to the hypertrophy of the gracile tubercle formed by the nucleus and fasciculus of the gracilis due to spheroid bodies deposition in sensory nuclei of the medulla [10]. The predominant MRI abnormalities were diffuse involvement of white matter (4/5;80%) in the DP subtype. Mineralization of substantia nigra and putamen was noted in a single patient with an EOP phenotype. The two patients with complex dystonia lacked any signature MRI involvement except for non-specific cerebellar atrophy (Figure 1, Table 1).

#### Ancillary investigations

Electroencephalogram (EEG) performed in 6 patients showed a generalized slowing in the theta range in 2 patients (Patient-8 & 9), focal interictal discharges in 2 patients (Patient-12 & 19) and a normal EEG in the remaining 2 patients. Neuropathy was documented in 3 patients (3/26;11.5%; Patient-2, 5 & 15), of which 2 had sensorimotor axonopathy (Patient-2 & 15) and a single patient had motor axonopathy (Table 1).

#### Management

A levodopa/carbidopa combination was administered in 14 patients with parkinsonism and/or dystonia. Twelve patients had at least 33% improvement either subjectively or objectively. Dopa-induced choreiform dyskinesia was noted in 10 out of 11 patients. Other symptomatic medications were administered, such as baclofen, clonazepam, tetrabenazine and trihexyphenidyl. None of the patients underwent deep brain stimulation (DBS) surgery (Table 1).

#### Genetic analysis

**Figure 2:**
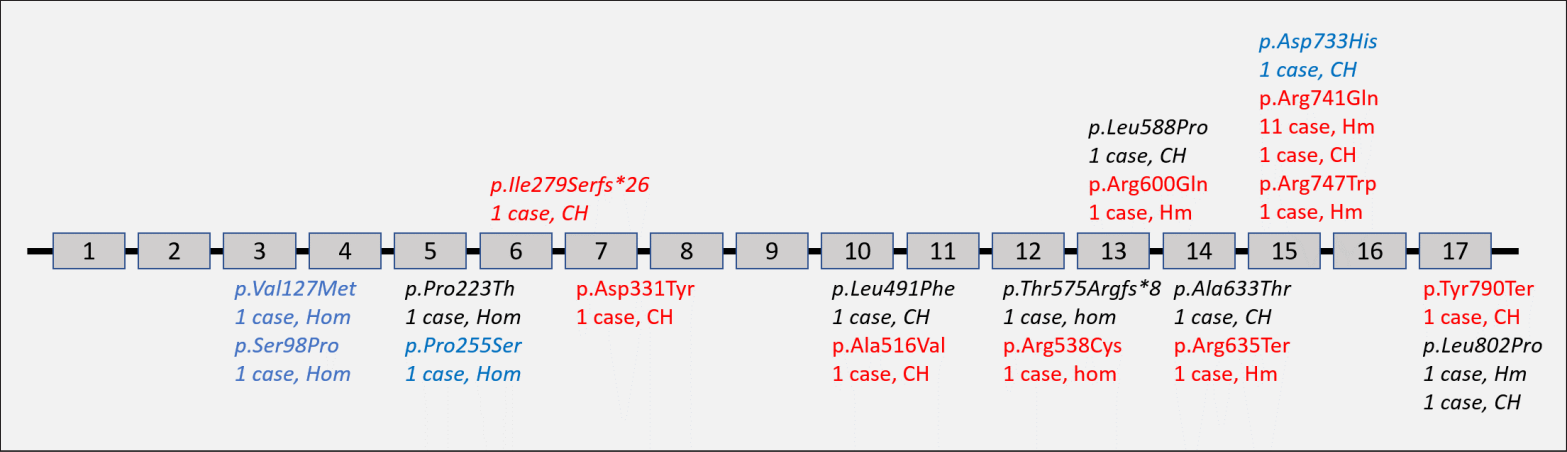
**Overview of the variants identified in this cohort.** Image depicting the location of the identified variants in the *PLA2G6* gene (Transcript ID: NM_003560.4). Novel variants are in italics, pathogenic variants are in red, likely pathogenic variants are in black and variant of uncertain significance are in blue.

Exome sequencing (Figure 2, Table 2) revealed biallelic disease-causing variants in the *PLA2G6* gene in all patients in homozygous state in 21 (21/26;80.8%) and compound heterozygous state in the remaining 5 patients (5/26;19.2%). Among patients with homozygous variants, missense variants were noted in 19 patients, while the remaining two patients had truncating variants (Stop-gain: 1 and frameshift: 1). The patients with homozygous truncating variants had the severe INAD phenotype. In five patients with compound heterozygous variants, three patients had missense/missense configuration and one patient each had missense/stop-gain and missense/frameshift configuration. The c.2222G>A;p.Arg741Gln variant was the most commonly identified variant in our cohort, which was identified in 12 patients (9 families). This variant has been previously reported and is predominantly seen in Asian subpopulations [1]. The next common variant was a novel c.2405T>C;p.Leu802Pro missense variant, which was observed in two patients. It was identified in homozygous state in one patient with EOP and in compound heterozygous configuration with a previously reported pathogenic stop-gain variant in another patient with cHSP phenotype.

**Table 2:** Details of disease-causing variants (Transcript ID: NM_003560.4).


FAMILY	PATIENT NO	AGE (YEARS)	GENDER	AAO (YEARS)	FAMILY HISTORY	CONSANGUINITY	FIRST SYMPTOM	CLINICAL PHENOTYPE	EXON	c.DNA	PROTEIN CHANGE	CONSEQUENCE	ACMG CRITERIA	ZYGOSITY	NOVEL

1	1	22	F	19.5	–	III	Slowness of ADL	DP	15	c.2239C>T	p.Arg747Trp	Missense	Pathogenic (PS3PP5PM1PM2PP2PP3)	Hm	–

2	2	34	F	26	+	III	Behavioural changes	DP	15	c.2222G>A	p.Arg741Gln	Missense	Pathogenic (PS3PP5PM1PM2PM5PP2)	Hm	–

3	3	1.9	M	0.5	+	No	Developmental regression	INAD	12	c.1723delT	p.Thr575 Argfs*8	Frameshift	Likely pathogenic (PVS1PM2)	Hm	+

4	4	29	F	15	+	No	Walking difficulty and leg posturing	DP	5	c.763C>T	p.Pro255Ser	Missense	VUS-LP (PM2PP2PP4)	Hm	+

5	5	1.9	M	1.4	–	III	Developmental regression	INAD	12	c.1612C>T	p.Arg538Cys	Missense	Pathogenic (PM5PS3PM1PM2PP2PP3PP5)	Hm	–

6	6	29	M	12	+	II	Cognitive decline	DPM	15	c.2222G>A	p.Arg741Gln	Missense	Pathogenic (PS3PP5PM1PM2PM5PP2)	Hm	–

6	7	24	F	12	+	II	Cognitive decline	DPM	15	c.2222G>A	p.Arg741Gln	Missense	Pathogenic (PS3PP5PM1PM2PM5PP2)	Hm	–

7	8	21	M	20	–	No	Speaking difficulty	cHSP	3	c.292T>C	p.Ser98Pro	Missense	VUS–LP (PM2PP2PP4)	Hm	+

8	9	8	M	2	–	No	Slurring of speech	ANAD	10	c.1547C>T	p.Ala516Val	Missense	Pathogenic (PM1PM2PP2PP3PP5)	CH	–

									10	c.1471C>T	p.Leu491Phe	Missense	Likely pathogenic (PM1PM2PP2PP3)	CH	+

9	10	7	F	4	–	No	Walking difficulty	ANAD	15	c.2222G>A	p.Arg741Gln	Missense	Pathogenic (PS3PP5PM1PM2PM5PP2)	CH	–

									13	c.1763T>C	p.Leu588Pro	Missense	Likely pathogenic (PM1PM2PP2PP3)	CH	+

10	11	28	M	25	–	No	Slowness of ADL	EOP	15	c.2197G>C	p.Asp733His	Missense	Likely pathogenic (PM2PP2PP3PP4)	CH	+

									14	c.1897G>A	p.Ala633Thr	Missense	Likely pathogenic (PM1PM2PM5PP2PP3)	CH	+

11	12	2	F	1	–	II	Regression of milestones	INAD	5	c.667C>A	p.Pro223Thr	Missense	Likely pathogenic (PM2PM5PP3PP4)	Hm	+

12	13	26	F	16	+	II	Toe walking	cHSP	15	c.2222G>A	p.Arg741Gln	Missense	Pathogenic (PS3PP5PM1PM2PM5PP2)	Hm	–

	14	32	M	20	+	II	Ataxia	cHSP	15	c.2222G>A	p.Arg741Gln	Missense	Pathogenic (PS3PP5PM1PM2PM5PP2)	Hm	–

13	15	3.5	F	2	–	III	Regression of milestones	INAD	13	c.1799G>A	p.Arg600Gln	Missense	Pathogenic (PM1PM2PM5PP2PP3PP5)	Hm	–

14	16	5	M	3	–	No	Regression of milestones	ANAD	3	c.379G>A	p.Val127Met	Missense	VUS–LP (PM2PP2PP4)	Hm	+

15	17	29	M	14	–	No	Behavioural issues	DP	6	c.835delA	p.Ile279SerfsTer26	Frameshift	Pathogenic (PVS1PM2)	CH	+

									7	c.991G>T	p.Asp331Tyr	Missense	Pathogenic (PM1PM2PM5PP2PP3PP5)	CH	–

16	18	36	F	10	–	No	Toe walking and running since childhood	DP	15	c.2222G>A	p.Arg741Gln	Missense	Pathogenic (PS3PP5PM1PM2PM5PP2)	Hm	–

17	19	23	M	18	+	II	Left leg dragging followed by walking difficulty	cHSP	15	c.2222G>A	p.Arg741Gln	Missense	Pathogenic (PS3PP5PM1PM2PM5PP2)	Hm	–

18	20	28	M	24	+	No	Slowness in daily activity	DPM	15	c.2222G>A	p.Arg741Gln	Missense	Pathogenic (PS3PP5PM1PM2PM5PP2)	Hm	–

18	21	31	M	28	+	No	Memory impairment	DPM	15	c.2222G>A	p.Arg741Gln	Missense	Pathogenic (PS3PP5PM1PM2PM5PP2)	Hm	–

19	22	30	F	28	–	Yes	Slowness of ADL	EOP	17	c.2405T>C	p.Leu802Pro	Missense	VUS-LP (PM2PM3PP2PP4)	Hm	+

20	23	2	M	1.5	–	II	Developmental regression	INAD	14	c.1903C>T	p.Arg635Ter	Stop gain	Pathogenic (PVS1PM2PP5)	Hm	–

21	24	22	F	22	+	II	Posturing and tremulousness of head and right upper limb	cDYT	15	c.2222G>A	p.Arg741Gln	Missense	Pathogenic (PS3PP5PM1PM2PM5PP2)	Hm	–

21	25	20	M	11	+	II	Posturing and tremulousness of head and right upper limb	cDYT	15	c.2222G>A	p.Arg741Gln	Missense	Pathogenic (PS3PP5PM1PM2PM5PP2)	Hm	–

22	26	15	M	5	–	No	Walking difficulty, stiff leg	cHSP	17	c.2370T>G	p.Tyr790Ter	Stop gain	Pathogenic (PVS1PS3)	CH	–

									17	c.2405T>C	p.Leu802Pro	Missense	Likely pathogenic (PM2PM3PP2PP4)	CH	+


+: Yes; –: No. AAO: Age at onset; ACMG: American College of Medical Genetics; ADL: Activity of daily living; ANAD: Atypical neuroaxonal dystrophy; cDNA: complementary Deoxyribonucleic acid; cDYT: Complex dystonia; CH: Compound heterozygous; cHSP: complicated Hereditary spastic paraparesis; DP: Dystonia parkinsonism; DPM: Dystonia parkinsonism myoclonus; EOP: Early onset Parkinsonism; F: Female; Hm: Homozygous; INAD: Infantile Neuroaxonal Dystrophy; M: Male; PLAN: PLA2G6-associated neurodegeneration; VUS-LP: Variant of uncertain significance probably likely pathogenic.

In total 19 unique variants were identified, of which 15 were missense variants, two were stop-gain and the remaining two were frameshift variants (Figure 2). Among these 19 unique variants, 11 variants were novel. Of these 11 novel variants, 9 were missense variants (c.292T>C;p.Ser98Pro, c.379G>A;p.Val127Met, c.667C>A;p.Pro223Thr, c.763C>T;p.Pro255Ser, c.1471C>T;p.Leu491Phe, c.1763T>C;p.Leu588Pro, c.1897G>A,p.Ala633Thr, c.2197G>C;p.Asp733His and c.2405T>C;p.Leu802Pro) and two were frameshift variants (c.835delA;p.Ile279SerfsTer26 and c.1723delT;p.Thr575 ArgfsTer8). Of these, as per ACMG criteria, eight variants could be classified as pathogenic or likely pathogenic, while three variants (c.292T>C;p.Ser98Pro, c.379G>A;p.Val127Met and c.763C>T;p.Pro255Ser) could be only classified as a variant of uncertain significance (VUS) at best. However, as per the recent scoring update to ACMG criteria, these variants could be reclassified as VUS more likely to be pathogenic than benign (VUS-LP) [9]. One novel variant (c.2405C>T;p.Leu802Pro) was seen in two patients, in homozygous state in a patient with EOP and in compound heterozygous *trans* configuration with a previously reported pathogenic stop-gain variant in a patient with cHSP owing to which it could be classified to likely pathogenic variant.

### Case vignettes

*Patient-13:* A 26-year-old previously healthy female with consanguineous parentage, normal birth and developmental history presented with a complex neurological illness spanning 10 years (Video 1). Initially, she noticed walking difficulty with stiffness in limbs and occasional toe walking. In addition, she had frequent falls and inappropriate smiling. Her elder brother also had walking difficulty for 12 years duration, along with inappropriate smiling. On examination (Video 1), both patients had mild upgaze restriction that improved on vestibulo-ocular maneuverer, pseudobulbar affect, spasticity, and brisk deep tendon reflexes in all four limbs with extensor plantar response bilaterally and cerebellar abnormality. MRI of the proband revealed mineralization of basal ganglia and substantia nigra with cerebellar and cortical atrophy. Exome sequencing revealed a homozygous pathogenic missense variant (c.2222G>A;p.Arg741Gln). The siblings were treated symptomatically with baclofen and physiotherapy.

**Video 1:** **Video of Patient-13 with complicated Hereditary Spastic Paraparesis.** Video of Patient-13 demonstrating pseudobulbar affect, mild dysarthria, mild clumsiness in hands, incoordination in left upper limb and bilateral lower limbs with spastic-ataxic gait. In addition, the patient had spasticity and brisk deep tendon reflexes in all four limbs with bilateral extensor plantar response (not shown in the video). Exome sequencing revealed homozygous c.2222G>A;p.Arg741Gln pathogenic missense variant. The video was taken after written informed consent was obtained for video recording, and publication in print and online.

*Patient-7:* A 24-year-old female born to consanguineous parentage with normal birth and developmental history presented with a twelve-year history of progressive cognitive impairment, followed by tremulousness in upper limbs, slowness, abnormal posturing and walking difficulty. In addition, the patient had a behavioral abnormality in the form of anger outbursts, crying spells, low mood and anxiety. On examination (Video 2), the proband had parkinsonism, rest tremor, facial predominant myoclonus, pyramidal signs, generalized dystonia and cerebellar involvement. MRI showed diffuse cortical and cerebellar atrophy with basal ganglia mineralization. Exome sequencing revealed a previously reported pathogenic homozygous missense variant (c.2222G>A;p.Arg741Gln). She was treated with levodopa with a good response but developed disabling choreiform dyskinesia. Her elder brother also had similar cognitive, behavioral, and motor symptoms of 12 years duration and, on examination, had parkinsonism, rest tremor, dystonia, pyramidal signs, myoclonus, and cerebellar abnormality. The rest tremor persisted in an outstretched position as well albeit with similar severity, and in the presence of cerebellar signs and distal hand dystonia, it can be a component of rubral tremor rather than parkinsonian rest tremor. Sanger sequencing in the brother confirmed the presence of c.2222G>A;p.Arg741Gln in homozygous state. He also responded to levodopa but with disabling dyskinesia.

**Video 2:** **Video of Patient-7 with dystonia-parkinsonism-myoclonus phenotype.** Video of Patient-7 demonstrating reduced facial expression, left more than right rest tremor, and distal appendicular predominant generalized dystonia. Post-levodopa, the patient developed generalized choreiform dyskinesia with partial improvement in parkinsonism and dystonia. In addition, the patient had facial predominant perioral action-induced myoclonus and pyramidal signs (not shown in the video). Exome sequencing revealed homozygous c.2222G>A;p.Arg741Gln pathogenic missense variant. The video was taken after written informed consent was obtained for video recording, and publication in print and online.

*Patient-25:* A 20-year-old male born to consanguineous parentage with normal birth and developmental history presented with an eight-year history of head and right upper limb posturing and tremulousness, alongside intellectual disability (Video 3). He demonstrated upgaze restriction, ataxic dysarthria, and generalized dystonia, with normal gait and brisk reflexes. MRI also revealed cerebellar atrophy. Exome sequencing revealed a previously reported pathogenic homozygous missense variant (c.2222G>A;p.Arg741Gln). The neck dystonia was associated with axial paroxysms of jerky dystonia commonly seen in patients with myoclonus dystonia (DYT-*SGCE*). However, there were no significant variant identified in *SGCE* gene. He did not respond to levodopa therapy and was put on clonazepam and baclofen for dystonia. His elder sibling, 22-year-old girl also had similar symptoms but of only 4 months duration. Examination revealed upgaze restriction, generalized dystonia, appendicular ataxia, and brisk reflexes, with a dystonic gait. MRI showed cerebellar atrophy. Sanger sequencing in the sister confirmed the presence of homozygous c.2222G>A;p.Arg741Gln variant. Her dystonia partially improved with levodopa therapy.

**Video 3:** **Video of Patient-25 with complex dystonia.** Video of Patient-25 demonstrating cervical predominant generalized dystonia with left torticollis with frequent spasmodic retrocollis, mild dystonia in outstretched hands with dystonic right thumb tremor, and truncal tilt to right on walking. In addition, the patient had mild cerebellar signs and brisk deep tendon reflexes. Exome sequencing revealed homozygous c.2222G>A;p.Arg741Gln pathogenic missense variant. The video was taken after written informed consent was obtained for video recording, and publication in print and online.

## Discussion

The current study, illustrating 26 patients of PLAN, represents the largest single-center series in the medical literature so far. The spectrum of the presentation was diverse, where 7 different clinical phenotypes were identified encompassing INAD, aNAD, DP, DPM, EOP, complex dystonia, and cHSP. Compared with the MDSgene [11] cohort that comprises 161 patients, our study population had male preponderance (57.7% vs 44.3%), higher AAO [13 (IQR 2.7–20.5) vs 7 (2–24) years], and a lower proportion of global developmental delay (34.6% vs 50.9%). Symptoms at onset were comparable except for a higher prevalence of developmental regression (23.1% vs 13.6%), and bradykinesia (15.4% vs 5.6%) (Table 3). Our cohort exhibited a lower prevalence of cognitive decline (46.2% vs 90.1%), strabismus (15.4% vs 78.8%), optic atrophy (3.8% vs 94.0%), nystagmus (11.5% vs 91.4%), gaze restriction (42.3% vs 94.7%), dysarthria (42.3% vs 94.7%), rigidity (46.2% vs 82.2%) compared to the MDSgene cohort. With regards to the extrapyramidal manifestations, a comparatively lower prevalence of cerebellar signs (53.8% vs 84.6%), tremors (26.9% vs 83.3%), parkinsonism (42.3% vs 69.6%), and drug-induced dyskinesia (38.5% vs 73.2%) were reported compared to the world literature. However, the proportion of patients with dystonia was comparable with the MDSgene cohort (53.8% vs 57.6%). Of note, owing to the review nature of the study, the MDSgene cohort had a significant proportion of missing data and mainly focused on dystonia and/or parkinsonism phenotype, which could partly explain the difference between our cohort and the MDSgene cohort.

**Table 3:** Table comparing clinical features between our cohort and the MDSgene cohort.


	CURRENT COHORT (n = 26) (%)	MDS GENE COHORT (n = 161) (%)*

Gender		

*Male*	15	62

*Female*	11	78

*Missing data*	0	21

Age at Onset, Year (median, IQR)	13.0 (2.7–20.5)	7 (2–24)^#^

Global Developmental Delay	9 (34.6)	28/55 (50.9)

**Symptoms at the onset**		

*Developmental regression*	6 (23.1)	12/88 (13.6)

*Behavioral symptoms*	2 (7.7)	9/88 (10.2)

*Dystonia*	2 (7.7)	1/88 (1.1)

*Gait disturbances*	7 (26.9)	6/88 (6.8)

*Bradykinesia*	4 (15.4)	5/88 (5.6)

*Dysarthria*	2 (7.7)	1/88 (1.1)

**Symptomatology**		

Behavioral changes^&^	6 (23.1)	–

Psychosis	4 (15.4)	18/35 (51.4)

Anxiety	2 (7.7)	9/20 (45.0)

Depression	2 (7.7)	17/35 (48.6)

Cognitive decline	12 (46.2)	82/91 (90.1)

Strabismus	4 (15.4)	41/52 (78.8)

Epilepsy	4 (15.4)	34/66 (51.5)

Optic atrophy	1 (3.8)	47/52 (94.0)

Nystagmus	3 (11.5)	64/70 (91.4)

Gaze restriction	11 (42.3)	18/19 (94.7)

Dysarthria	11 (42.3)	33/44 (75.0)

Tone		

*Rigidity*	12 (46.2)	60/73 (82.2)

*Spasticity*	7 (26.9)	51/55 (92.7)

*Hypotonia*	5 (19.2)	55/55 (100)

Brisk muscle stretch reflex	17 (65.4)	44/71 (62.0)

Positive Babinski sign	12 (46.2)	118/121 (97.5)

Extrapyramidal features		

*Dystonia*	14 (53.8)	57/99 (57.6)

Generalized	12 (46.2)	6/44 (13.6)

Lower limb	1 (3.8)	12/38 (31.6)

*Parkinsonism*	11 (42.3)	55/79 (69.6)

*Tremor*	7 (26.9)	10/12 (83.3)

*Positive cerebellar signs*	14 (53.8)	44/52 (84.6)

*Myoclonus*	6 (23.1)	4/4 (100)

Gait disturbance	21 (80.8)	43/43 (100)

Dyskinesia	10 (38.5)	30/41 (73.2)


* Information for all 161 patients was not accessible; every variable exhibited missing data, resulting in a fluctuating denominator for each variable. The denominator does not include missing data and is only sum of positive and negative wherever data was available. ^#^ Missing data = 36 (31.3%). ^&^ Patients exhibited multiple behavioral symptoms, including depression, psychosis, and anxiety, either individually or in combination. IQR: Inter quartile range.

### PLA2G6-associated cHSP phenotype

Our study cohort of 5 patients of cHSP phenotype represents the second largest series on this unique phenotype in the medical literature (Table 4). Previously, there were only 4 publications that reported on cHSP phenotype [12,13,14,15]. The largest series of 6 patients by Koh et al. from Japan, had a female preponderance, high prevalence of cognitive decline (5/6), and cerebellar atrophy on neuroimaging (5/6) [13]. At par with the above phenotype, cognitive decline was observed in 4/5 (80%) of our patients, and all except one exhibited cerebellar signs. The unique features identified in our cohort included dysarthria (n = 3, 60%), dystonia (n = 3, 60%, generalized in n = 2, 40%), epilepsy (n = 2, 40%), and gaze restriction (n = 3, 60%).

**Table 4:** Review of the patients of PLAN with cHSP phenotype.


	CURRENT STUDY (n = 5)	CHENG ET AL. 2022 (n = 1)	KOH ET AL. 2018 (n = 6)	CHEN ET AL. 2018 (n = 2)	OZES ET AL. 2017 (n = 2)

Gender (Male: Female)	4:1	Female	2:4	1:1	NA

Age, Year (median, IQR)	23.0 (18.0–29.0)	13	NA	26 (20–32)	NA

AAO, Year (median, IQR)	18.0 (10.5–20.0)	6	Infantile = 2 1 year = 2 10 years = 1 66 years = 1	19 (7–31)	9year 21year

DOI, month (median, IQR)	10.0 (3.0–11.0)	7 years	NA	7 (1–13) Years	NA

Consanguinity (n, %)	3 (60.0)	NA	NA	NA	NA

Positive family history n, %)	3 (60.0)	0	NA	1 (50)	NA

Developmental Delay	1 (20.0)	NA	NA	1 (50)	NA

Dysmorphism	0	0	NA	0	NA

**Symptoms at the onset**					

Developmental regression	0	Yes	NA	0	0

Cognitive decline	0	Yes	2	1 (50)	0

Behavioural symptoms	0	0	0	0	0

Abnormal Posturing	0	0	0	0	0

Walking difficulty	4 (80.0)	Yes	4	1 (50)	Yes

Slowness	0	0	0	0	0

Change in speech	1 (20.0)	0	0	0	0

**Symptomatology**					

Behavioural changes	3 (60.0)	Yes	NA	1 (50)	Yes (1/2, 50%)

Cognitive decline	4 (80.0)	Yes	5	1 (50)	0

Drooling of saliva	1 (20.0)	0	NA	NA	0

Pseudobulbar affect	2 (40.0)	0	NA	NA	0

Strabismus	0	0	NA	0	0

Optic atrophy	0	0	NA	0	0

Nystagmus	0	0	NA	0	0

Epilepsy	2 (40.0)	0	NA	0	0

Gaze restriction	3 (60.0)	0	NA	0	0

Speech abnormality	3 (60.0)	0	NA	1 (50)	2 (100)

**Tone**					

Spasticity	5 (100.0)	1 (100)	6 (100)	2 (100)	2 (100)

Brisk muscle stretch reflex	5 (100.0)	1 (100)	6 (100)	2 (100)	2 (100)

Positive Babinski sign	4 (80.0)	1 (100)	6 (100)	2 (100)	2 (100)

**Extrapyramidal features**			2 (33.3)		

Dystonia	3 (60.0)	1 (100)	NA	2 (100)	0

Generalized	2 (40.0)	–	NA	NA	–

Lower limb only	0	–	NA	NA	–

Finger only	1 (20.0)	–	NA	NA	–

Parkinsonism	0	0	NA	NA	0

Tremor	1 (20.0)	0	NA	NA	0

Contractures	3 (60.0)	0	NA	NA	0

Positive cerebellar signs	3 (60.0)	0	3 (50)	2 (100)	1 (50)

**Gait** Dystonic Ataxic Spastic	0 2 (40.0) 2 (40.0)	0 0 1 (100)	NA	0 0 2 (100)	0 0 2 (100)

Myoclonus	2 (40.0)	0	NA	NA	0

Abnormal EEG	1/2 (20.0)				

Generalized slowing	0	NA	NA	NA	NA

Focal IEDs	1/2 (20.0)				

Normal	1/2 (50.0)				

Neuropathy	0	NA	NA	NA	NA

Response to Levodopa	0	NA	NA	NA	1 (50)

Dyskinesia	0	NA	NA	NA	0

**Neuroimaging**	n = 4	n = 1	n = 6	n = 2	n = 2

*Mineralization*	4 (100)	1 (100)	2 (33.3)	1 (50)	2 (100)

Caudate	3 (75.0)	0	0	0	0

Putamen	3 (75.0)	0	0	0	0

GPi	3 (75.0)	1 (100)	2 (33.3)	1 (50)	2 (100)

Substantia nigra	4 (100.0)	1 (100)	0	1 (50)	1 (50)

*Atrophy*					

Caudate	1 (25.0)	0	0	0	0

Cerebral	3 (75.0)	1 (100)	0	0	0

Corpus callosum	0	0	0	0	0

Optic nerve	0	0	0	0	0

Cerebellar	4 (100)	1 (100)	5 (83.3)	2 (100)	0

White matter signal changes	3 (75.0)	0	0	0	0

Claval hypertrophy	2 (50.0)	0	0	0	1 (50)

Genetic analysis	c.2222G>A (n = 3, 2 families) c.2370T>G (n = 1) c.292T>C (n = 1)	c.1427 + 2T>A	c.517C>T/c.1634 A>G (n = 2, same family) c.662T>C/c.991G>T (n = 1) c.1187-2A>G/c.1933C>T (n = 2, same family) c.1904G>A (n = 1)	c.1511C>T/ c.1117G>A (n = 1) c.991G>T/ c.1982C>T (n = 1)	c.1786C>T (n = 2, same family)


AAO: Age at onset; cHSP: Complicated Hereditary spastic paraparesis; DOI: Duration of illness; IQR: Inter quartle range; NA = Not available.

With regards to neuroimaging, cerebellar atrophy and mineralization in substantia nigra were seen in all patients with available brain MRI (n = 4). There were 3 patients (75%) that were detected to have mineralization involving caudate, putamen, and globus pallidus interna (GPi). Unlike previous reports, white matter signal changes were reported in 75% of patients (n = 3/4), while claval hypertrophy was reported in 2 patients. Interestingly, the most common genetic variant: c.2222G>A identified in 3 patients (60%), was not previously reported with *PLA2G6*-related complicated cHSP phenotype. The difference in clinical phenotypes and neuroimaging findings as compared to the previous patients could be accounted for by the differences in the genotypic spectrum of the identified variants in the *PLA2G6* gene.

### PLA2G6-associated phenotypes related to c.2222G>A variant

The c.2222G>A is the most common genetic variant identified in our cohort of PLAN (34.6%). Previously 19 patients have been reported in the literature on this genetic variant (Table 5) [1,2,16,17,18,19,20,21,22]. Amongst the patients reported previously, a large proportion of them belong to Asian ancestry, and of these, 12 patients either are from India or have an Indian ancestry [1,17,18,19,20,21]. Our study contributes to the largest single-center series of patients identified with c.2222G>A variant. The demographic parameters of this subgroup were at par with previous publications, exhibiting a nearly equal sex ratio, similar AAO (Median: 28 years; IQR: 23.5-33 years), a similar prevalence of family history (77.8% vs 78.9%), and consanguinity (66.7% vs 72.2%). The unique attributes that were recognized include the occurrence of cHSP phenotype (n = 3, 33.3%), DPM (n = 3, 33.3%), and aNAD (n = 1, 11.1%), which were not known previously. While previous studies have suggested a much greater prevalence of DP (n = 16/19, 84.6%), only 2 of our patients (22.2%) displayed such a phenotype. A recent report demonstrated a remarkable response to bilateral subthalamic nuclei DBS in a patient diagnosed with PLAN carrying this specific variant in the homozygous state [21]. At three months follow-up, the patient experienced significant relief from dyskinesia (>90%), improvement in OFF-state (UPDRS-III score 61 to 16), and reduction in levodopa-equivalent daily dose (1050 mg to 275 mg). This emphasizes the crucial importance of identifying this variant in patients clinically suspected of having this condition [1]. However, the long-term sustenance of the benefit needs to be studied. There are two more reports of STN-DBS in patients with PLAN but with different variants. In the report by Wirth et al., the patient carried compound heterozygous variants (c.109C>T;p.Arg37X and c.232G>T;p.Ser774Iso) in *PLA2G6* gene and noted around 70% improvement in UPDRS-III OFF state score at one-year follow-up [23]. In the other report by Choi et al. (conference abstract), the authors mention that the siblings carrying compound heterozygous variants (c.359G>A;p.Trp120Ter and c.1742G>A;p.Arg581Gln) in *PLA2G6* gene had an excellent response to bilateral STN-DBS [24]. However, no further details are available. In view of only a few case reports, one needs to be cautious with respect to the long-term benefits of STN-DBS in patients with PLAN.

**Table 5:** Review of the patients of PLAN with homozygous c.2222G>A variant.


VARIABLE (N, % UNLESS SPECIFIED)	CURRENT STUDY (N = 12, 8 FAMILIES)	REVIEW OF LITERATURE (N = 19)

**Clinical**		

Phenotype	cHSP = 3; DPM = 4; DP = 2; ANAD = 1; cDYT = 2	DP = 16 EOP = 3

Gender (Male: Female)	6:6	9:10

Age, Year (median, IQR)	27.0 (22.25–31.75)	28.0 (24.0–33.0)

AAO, Year (median, IQR)	17.0 (11.25–23.5)	22.0 (16.0–25.0)

DOI, month (median, IQR)	12.0 (3.25–12)	6.5 (3.0–11.3)

Consanguinity	5/8 families (62.5)	13 (72.2)

Positive family history	10 (83.3)	15 (78.9)

Developmental Delay	3 (25)	1/10 (10)

Dysmorphism	0	–

Symptoms at the onset		*

*Cognitive decline*	3 (25)	1 (5.6)

*Behavioural symptoms*	1 (8.3)	12 (66.7)

*Abnormal Posturing*	2 (16.7)	0

*Walking difficulty*	5 (41.7)	3 (16.7)

*Parkinsonism*	1 (8.3)	2 (11.1)

Symptomatology		

*Behavioural changes*	5 (41.7)	17/18 (94.4)

*Cognitive decline*	6 (50)	18/18 (100)

*Pseudobulbar affect*	3 (25)	10/16 (62.5)

*Speech abnormality*	3 (25)	10/14 (66.7)

Optic atrophy	0	2/2 (100)

Gaze restriction	8 (66.7)	–

Tone		

*Rigidity*	6 (50)	16 (88.9)

*Spasticity*	3 (25)	2 (11.1)

*Hypotonia*	1 (8.3)	NA = 1

Brisk muscle stretch reflex	10 (83.3)	16/16 (100)

Positive Babinski sign	6 (50)	7/15 (46.7)

Extrapyramidal features		

Dystonia	9 (75)	14 (73.7)

Generalized	8 (66.7)	–

Lower limb only	1 (8.3)	–

Finger only	0	–

*Parkinsonism*	6 (50)	19 (100)

*Tremor*	6 (50)	10/18 (55.6)

*Positive cerebellar signs*	6 (50)	2/16 (12.5)

Myoclonus	5 (41.7)	6/17 (35.3)

Contractures	6 (50)	

Gait		

*Dystonic*	6 (50)	–

*Ataxic*	1 (8.3)	–

*Spastic*	3 (25)	

Response to Levodopa	1 (11.1)	18/18 (100)

Levodopa-induced Dyskinesia	4 (44.4)	12/18 (66.7)

**Neuroimaging**		

Mineralization		

*Caudate*	6/11 (54.6)	2/16 (12.5)

*Putamen*	5/11 (45.5)	3/16 (18.8)

*GPi*	4/11 (36.4)	3/16 (18.8)

*Substantia nigra*	5/11 (45.5)	2/16 (12.5)

Atrophy		

Caudate	4/11 (36.4)	–

Cerebral	6/11 (54.6)	14/18 (77.8)

Frontal predominant	3/11 (27.3)	4/18 (22.2)

Optic nerve	0	2/16 (12.5)

Cerebellar	10/11 (90.9)	11/18 (61.1)

White matter signal changes	5/11 (45.5)	1/18 (5.6)


cHSP: Complicated Hereditary spastic paraparesis, DPM: Dystonia parkinsonism myoclonus. DP: Dystonia parkinsonism, EOP: Early onset Parkinsonism, ANAD: Atypical neuroaxonal dystrophy. *Based on the availability of the information, denominators are variable, in this case data of 15 patients were available for analysis. NA = Not available.

## Limitation

Our study has several limitations. Owing to the retrospective nature, the investigations are not uniform, and there is a possibility of missing clinical information. A formal cognitive assessment of patients with cognitive decline was not done in our patients. Additionally, a dedicated severity scale for parkinsonism and dystonia was not uniformly available. Previous studies have demonstrated the utility of tissue diagnosis that may aid in identifying axonal spheroids. However, none of our patients had any tissue diagnosis. The absence of longitudinal follow-up was one of the drawbacks.

## Conclusion

The current study adds 26 new patients of PLAN showcasing a diverse range of clinical manifestations, potentially constituting the most extensive series of PLAN patients published in the medical literature. Among these patients, 12 new instances of PLAN associated with the recently recognized c.2222G>A variant have been identified, a variation previously noted to exhibit a predisposition among individuals of Asian descent. Additionally, it broadens the spectrum of neuroimaging findings related to cHSP associated with PLAN. We observed three patients displaying a cHSP phenotype in patients with c.2222G>A variant. Furthermore, our study revealed 11 novel variants in the *PLA2G6* gene, expressed both in homozygous and compound heterozygous states.
